# Comparing the gut microbiome of obese, African American, older adults with and without mild cognitive impairment

**DOI:** 10.1371/journal.pone.0280211

**Published:** 2023-02-24

**Authors:** Andrew McLeod, Beatriz Penalver Bernabe, Yinglin Xia, Jennifer Sanchez-Flack, Melissa Lamar, Linda Schiffer, Karla Castellanos, Giamila Fantuzzi, Pauline Maki, Marian Fitzgibbon, Lisa Tussing-Humphreys

**Affiliations:** 1 Department of Kinesiology and Nutrition, University of Illinois Chicago, Chicago, Illinois, United States of America; 2 Institute for Health Research and Policy, University of Illinois Chicago, Chicago, Illinois, United States of America; 3 Department of Biomedical Engineering, University of Illinois Chicago, Chicago, Illinois, United States of America; 4 Department of Medicine, University of Illinois Chicago, Chicago, Illinois, United States of America; 5 Department of Pediatrics, University of Illinois Chicago, Chicago, Illinois, United States of America; 6 University of Illinois Cancer Center, University of Illinois Chicago, Chicago, Illinois, United States of America; 7 Rush Alzheimer’s Disease Center, Rush University, Chicago, Illinois, United States of America; 8 Departments of Psychology and Psychiatry, University of Illinois Chicago, Chicago, Illinois, United States of America; University of Alabama at Birmingham, UNITED STATES

## Abstract

Those with mild cognitive impairment (MCI), a precursor to dementia, have a gut microbiome distinct from healthy individuals, but this has only been shown in healthy individuals, not in those exhibiting several risk factors for dementia. Using amplicon 16S rRNA gene sequencing in a case-control study of 60 older (ages 55–76), obese, predominately female, African American adults, those with MCI (cases) had different gut microbiota profiles than controls. While microbial community diversity was similar between cases and controls, the abundances of specific microbial taxa weren’t, such as *Parabacteroides distasonis* (lower in cases) and *Dialister invisus* (higher in cases). These differences disappeared after adjusting for markers of oxidative stress and systemic inflammation. Cognitive scores were positively correlated with levels of *Akkermansia muciniphila*, a bacterium associated with reduced inflammation. Our study shows that gut microbial composition may be associated with inflammation, oxidative stress, and MCI in those at high risk for dementia.

## Introduction

In the United States (U.S.), dementia affects approximately 8.5% of adults over the age of 65 [1], costing an estimated $157-$215 billion annually, exceeding the U.S. health care expenditures of both heart disease and cancer [2]. Moreover, 16.6% of adults 65 years or older exhibit mild cognitive impairment (MCI) [3], an intermediary stage between normal cognition and dementia that can increase risk of dementia by almost three-fold [4, 5]. Several other risk factors for dementia have been studied, a major one being obesity, which alone increases risk of dementia by 60% [6]. Alarmingly, obesity prevalence is 38% in adults 65 and older [7] and has been increasing for the past two decades [8]. Race is also a risk factor for dementia with African American adults having a 64% higher risk of Alzheimer’s Disease (AD) compared to Caucasians (RR = 1.64, 95% CI = 1.35–2.00) [9], as well as sex, with females having a higher risk [10]. These observations suggest obese, African American women with MCI have a substantially higher risk of dementia compared to the general population.

An increasingly appreciated factor of neurodegenerative disorders, including dementia, may be the gut microbiota, the microscopic organisms of the intestines (e.g., bacteria, fungi, virus). There is bidirectional communication between the brain, gut, and gut microbial communities that has been coined the brain-gut-microbiota-axis [11, 12]. Age-related changes to the microbial structure of the gut that favor diminished bacterial diversity [13] and deficiencies in the production of key microbial metabolites, including the short chain fatty acid (SCFA) butyrate, may contribute to cognitive dysfunction in older adults [14]. In turn, these age-related dysfunctions of the gut microbiota may promote bacterial endotoxin translocation [15], further inflammation, and oxidative stress, all of which represent potential pathophysiological mechanisms of age-related cognitive decline and dementia [16–20].

The above suggests impaired cognition could be related to gut microbial structure and metabolism. However, only one study has analyzed the gut microbiome in those with MCI within the U.S. [21], but other important factors in MCI, such as obesity and race (e.g., African American), were not considered. Also, few studies have investigated in humans the potential pathophysiological mechanisms linking an altered gut microbiome to cognitive impairment.

The purpose of the current study was to characterize the gut microbiome of an obese group of predominately female African American adults with and without probable MCI and concurrently measure several key features linked to both the microbiome and cognition including lifestyle factors (e.g., smoking), markers of systemic bacterial endotoxin translocation, inflammation, and oxidative stress. We found differences in the gut microbiomes between those with and without probable MCI, and these differences were linked to markers of inflammation and oxidative stress.

## Materials and methods

### Study design, recruitment and participant selection

This case-control study utilized baseline data, i.e., information from participants at the time of enrollment, obtained from the Building Research in Diet and CoGnition (BRIDGE) study (NCT03129048), a prospective, randomized controlled trial comparing the effect of a Mediterranean diet (Med Diet) with and without weight loss on cognition among older obese adults. A description of the parent study design and methods and baseline characteristics of the full study population has been published [22, 23].

Briefly, participants were invited to be in the BRIDGE trial if they were obese (body mass index (BMI) between 30 and 50 kg/m^2^), and with an age between 55 and 85 years. Additionally, they were required to be relatively healthy, with no condition that would preclude them from participating in dietary interventions, exercise, or cognitive evaluations, excluding those with conditions such as uncontrolled diabetes, lack of ambulatory ability, or psychiatric disorders. Participants were also required to score 19 or above on the Montreal Cognitive Assessment (MoCA) [24], and not be adherent to a Med Diet based on an adapted dietary screener [25]. The University of Illinois Chicago Institutional Review Board reviewed and approved this study (IRB #2016–0258). All participants provided written informed consent prior to commencing the baseline visit.

For the current case-control study, cases and controls were selected from those enrolled in the BRIDGE study (n = 185) but that additionally had not taken antibiotics within 6 weeks of data collection and were willing to provide a stool sample (n = 133) [26]. From this sub-sample, cases and controls were identified where those scoring 19–25 on the MoCA were considered cases and those scoring 26–30 on the MoCA were considered controls [27]. 84 cases and 49 controls were identified. Cases and controls were then frequency matched on BMI (±2 units), age (±2 years), and sex. Not all cases could be matched to controls as some individuals had unique combinations of BMI, age, and sex. We were able to match a maximum of 30 cases to 30 controls, which was sufficient to meet our proposed sample size (see “*Sample Size Determination”* paragraph below). We did not match on race, as racial composition was almost the same between cases and controls (see Table 1).

**Table 1:** Participant characteristics stratified by case control status.

Variable	Cases (n = 30) (MoCA mean 22 (SD 1.7) range: 19–25)	Controls (n = 30) (MoCA mean 27 (SD 1.1) range: 26–30)	*p*-value
Age, years (mean (SD))	64.7 (5.7)	64.9 (5.7)	0.89
BMI, kg/m^2^ (mean (SD))	36.1 (3.8)	36.4 (4.1)	0.80
Female (n, %)	28, 93%	28, 93%	0.69
African American (n, %)	28, 93%	28, 93%	1.0
Any college education (n, %)	28, 93%	29, 97%	0.50
At least Associates degree (n, %)	17, 57%	22, 73%	0.085
College graduate (n, %)	12, 40%	19, 63%	0.035
<$40K annual household income (n, %)	17, 56%	17, 56%	1
Med Diet Score [46, 47] (range 0–55) (mean (SD))	34 (6)	33 (6)	0.53
Type 2 diabetes (n, %)	4, 13%	6, 20%	0.25
Depression score from CESD (mean (SD))	8.2 (6.7)	8.1 (6.2)	0.46
Sedentary time, hour/valid day (median (IQR))*	14.8 (2.1)	15.5 (1.7)	0.0497
Using microbiome-altering medications/supplements** (n, %)	9, 30%	7, 23%	0.56
Using cholesterol-lowering medications (n, %)	5, 17%	7, 23%	0.52
Using anti-inflammatory medications*** (n, %)	6, 20%	9, 30%	0.37
Using anti-hypertensive medications (n, %)	17, 57%	20, 67%	0.43
Currently smoking (n, %)	0, 0%	2, 6.7%	1.0
Systolic blood pressure, mmHg (mean (SD))	135 (19)	130 (17)	0.13
Diastolic blood pressure, mmHg (mean (SD))	83 (13)	79 (10)	0.054
Visceral Fat/Height, g/cm (median (IQR))	8.6 (3.8)	7.2 (3.1)	0.18

*Sedentary time was defined as <2000 counts per minute. Sedentary time was calculated as total number of 1-minute epochs of sedentary time divided total number of valid days worn. This value was divided by 60 to determine hours/day.

**Gut microbiome-altering medicines include probiotics, prebiotics, antihistamines, fiber supplements, and osmotic laxatives.

***Anti-inflammatory medication usage includes habitual use of inhaled steroids, oral prednisone and aspirin and short-term usage of NSAIDs or steroids 24 hours prior to blood draw.

*p*-values were determined for all continuous variables by a two-sample t-test, except for sedentary time and visceral fat/height, for which the Wilcoxon Rank Sum test was used. *p*-values were determined for all categorical variables by a chi-squared test. Abbreviations: BMI—Body Mass Index; CESD—Center for Epidemiological Studies–Depression; IQR—Interquartile Range; MCI—mild cognitive impairment; MoCA—Montreal Cognitive Assessment; SD—Standard Deviation.

## Data collection

### Stool and blood

Participants fasted for at least 8 hours prior to blood collection through venipuncture. Blood was centrifuged at 3,000 RPM at 4°C to generate plasma and serum and stored at -80°C until analysis. Participants collected stool at home 24–48 hours before the blood draw. Stool samples were stored at 4°C for 60–72 hours after stool production, aliquoted and then stored at -80°C until analysis.

### Stool genomic DNA extraction

Genomic DNA was extracted using DNeasy PowerSoil Kit (Qiagen, Valencia, CA) in combination with a bead-beating step using the FastPrep-24 System (MP Biomedicals).

### Genomic DNA library prep and sequencing

Genomic DNA was PCR-amplified with the Earth Microbiome Project primers CS1-515F and CS2-806R targeting the V4 regions of the 16S rRNA gene as previously described [28]. Briefly, extracted DNA from stool samples was mixed with both V4-targetting primers, CS1-515F and CS2-806R, followed by the addition of unique 10-base barcodes (Fluidigm, South San Francisco, CA; Item# 100–4876). MyTaq HS 2X mastermix (Bioline) was used during PCR-amplification. Negative controls were added during the extraction process to detect possible contamination occurring at this step. Separate negative controls were also added to the PCR-amplification process. Subsequently, adapting a prior published protocol [29], equal volume of samples were pooled and then filtered to remove DNA less than 300bp using an AMPure XP cleanup protocol (0.6X, vol/vol; Agencourt, Beckmann-Coulter). Purified DNA was then loaded onto an Illumina MiniSeq flow cell with a 20% phiX spike-in. If the number of reads per sample was unbalanced across samples, then the amplicons were re-pooled in volumes inversely proportional to their number of reads and purified using AMPure XP cleanup in the same manner as described above. The re-pooled libraries were loaded onto a Miniseq flow cell with a 20% phiX spike-in and sequenced (2x153 paired-end reads).

Sequences were quality filtered (Q≥35), and the first 20 nucleotides of the forward and reverse reads were trimmed. Sequences were then dereplicated and assigned an Amplicon Sequence Variant (ASV) using DADA2 with default parameters [30]. ASV taxonomy assignment up to genus level was obtained using the latest Silva database (v138) at 99% similarity [31], and species assignment was obtained using Silva v132 at 100% similarity [31]. ASVs were removed from down-stream analysis if they were present in the negative controls more than 3 times than in the participant samples, had less than 10 counts in all the participant samples, or had relative abundance less than 0.01%. ASV abundances were then normalized using cumulative sum scaling [32].

### Gut microbial structure analysis

To measure alpha diversity, we used the Chao1 [33], Shannon [34] and Inverse Simpson [35] indices. Beta diversity was measured using Jaccard [36], Bray-Curtis dissimilarity [37] and weighted/unweighted and normalized/non-normalized UniFrac distances [38] as implemented in the R package Phyloseq [39].

PERMANOVA in the R package vegan was used to determine differences between cases and controls for gut microbial alpha-diversity and beta-diversity metrics. Using the fitzig function in the *metagenomeSeq* [32] package in R, zero-inflated generalized linear models (GLM) were employed to identify taxa that were significantly associated with MCI status and MoCA score (treated as a continuous variable). We modeled only taxa that were present in a minimum number of participants. This number is called the least effective sample size and was calculated using the calculateEffectiveSamples function in *metagenomeSeq*. Models were adjusted for potential confounders (i.e., glucose, total cholesterol, systolic blood pressure (SBP), diastolic blood pressure (DBP), visceral fat, MedDiet score, physical activity, race, education, income, depression score, microbiome-altering medication use). Potential confounders that were significantly different at or below the alpha = 0.10 were included in the adjusted models. A more liberal alpha level was chosen given the exploratory nature of the study. FDR-adjusted *p* values were calculated using the Benjamini-Hochberg method to correct for all multiple comparisons [40].

### Butyryl-CoA CoA-transferase gene (*BcoA*) abundance

*BcoA* abundance was quantified using real time PCR (rt-PCR). The *BcoA* primers BCoATscrF and BCoATscrR and the synthetic DNA template standard (530 bp) were synthesized by Integrated DNA Technologies (IDT, WI), based on the method described by Louis, et al. [41]. After primer efficiency and optimization, samples were amplified by rt-PCR in triplicate using Fast SYBR^®^ Green Master Mix (Applied Biosystems, CA) and the ViiA7 rt-PCR System (Applied Biosystems, CA), under the following conditions: 2 min at 50°C, 2 min at 95°C, and 40 cycles of 1 s at 95°C, 20 s at 58°C, and 30 s at 72°C each with data acquisition at 72°C. Because the melt curve data from optimization produced ambiguous results, the rt-PCR products were viewed on 2% agarose E-Gel (Life Technologies, CA) to confirm presence of a single PCR product.

### Systemic inflammation and oxidative stress

High sensitivity C-reactive protein (hs-CRP), a common clinical marker of systemic inflammation, was assessed by Quest Diagnostics (Wood Dale, IL, USA) utilizing nephelometry (lower limit of quantitation = 0.2 mg/L). Oxidative stress was evaluated through measuring in duplicate two plasma F2-isoprostanes, prostaglandin-F2α (PGF2α) and 8-iso-prostaglandin-F2α (8-iso-PGF2α), using Ultra High-Performance Liquid Chromatography Mass Spectrometry/Mass Spectrometry. Standards were prepared in acetonitrile. Sample measurements were adjusted by a matrix factor used to compensate for signal suppression. Measurements were acquired with a Shimadzu (Kyoto, Japan) LCMS-8050 triple quadrupole mass spectrometer equipped with a Shimadzu Nexera UHPLC system. Measurements below the lower limit of detection (LOD) were given values of ½ the LOD [42].

### Endotoxin translocation

Lipopolysaccharide binding protein (LBP) was measured in duplicate in plasma via ELISA as the marker of bacterial endotoxin translocation (MilliporeSigma, St. Louis, MO). LBP has been used in several previous studies as an indirect marker of bacterial endotoxin translocation across the gut barrier [43, 44]. The intra-assay CV% for the current study was 3.6%.

### Glucose control and blood lipids

Hemoglobin A1c (HbA1c) was evaluated using participant whole blood at baseline at Quest Diagnostics (Wood Dale, IL) using immunoturbidity (CV% = 1.09). Fasting serum glucose was evaluated at baseline at Quest Diagnostics (Wood Dale, IL) using spectrophotometry (CV% = 1.506). A standard blood lipid panel of cholesterol and triglycerides was also performed by Quest Diagnostics (Wood Dale, IL) using fasting serum and spectrophotometry (total cholesterol CV% = 1.64, HDL cholesterol CV% = 2.8, triglyceride CV% = 1.7).

### Blood pressure

Diastolic and systolic blood pressure were measured in duplicate with the participant in a relaxed and seated position. An automatic blood pressure monitor was used (Omron HEM-907 (Lake Forest, IL)). The average of these two measurements is reported. Blood pressure is reported and analyzed because midlife (age 35–64) hypertension is a risk factor for Alzheimer’s disease (AD) [6].

### Anthropometrics and body composition

Height to the nearest 0.5 cm and body weight to the nearest 0.1 kg were measured in duplicate using a fixed stadiometer (Seca, Birmingham, UK) and digital scale (Tanita, Arlington Heights, IL), respectively. BMI was reported as kg/m^2^. Whole body composition was measured using dual-energy x-ray absorptiometry (DXA) with a Lunar iDXA machine (GE Healthcare, US). Central adiposity was estimated by the DXA software as the percentage of total mass that is android fat mass.

### Dietary intake

Dietary intake was measured with the Harvard Food Frequency Questionnaire (HFFQ) [45]. The HFFQ captures self-reported diet within the past 12 months, is semi-quantitative, with questions regarding frequency and serving sizes, and covers 131 food items. Trained staff administered the survey and the Channing Lab at Harvard University processed the HFFQs.

### Med Diet adherence

Med Diet adherence was defined by the score, first developed by Panagiotakos et al. [46], and later modified by Tangney et al. [47], for an urban U.S. Midwest population and was generated in the same manner as Karstens, et al. [48]. Briefly, each of the items on the HFFQ were categorized into one of 11 Med Diet score components. The 11 components were (1) non-refined grains, (2) potatoes, (3) fruit, (4) vegetables, (5) legumes and nuts, (6) fish, (7) olive oil, (8) alcohol, (9) red meat and processed meat, (10) poultry, and (11) full-fat dairy products. On each of these components a participant could score 0 to 5, with 0 representing minimum adherence and 5 representing maximum adherence. Thus, the Med Diet score fell within a range of 0–55. Higher consumption for components 1–7 resulted in higher component scores, but the reverse was true for components 9–11, with lower consumption resulting in higher component scores. Component 8, alcohol, was scored 0 for high or no consumption, with other levels of consumption in between varying proportionally with score.

### Physical activity

A wristworn accelerometer (ActigraphGT3X + monitor, Pensacola Florida) was used to provide an objective measure of physical activity [49] given low physical activity is a risk factor for dementia [6]. Participants were instructed to wear the accelerometer for 7 full days. Data were considered valid if the accelerometer was worn at least 4 days, for at least 10 hours each of those days. Sedentary time was determined using ActiLife v6.13.4 software (ActiGraph, Pensacola, FL), and was defined as <2,000 counts per minute as recommended by Kamada, et al. [50]. Sedentary time is represented as hours per valid day and was derived by dividing the total number of one-minute epochs per day by 60.

### Socio-demographic and health variables

These are self-reported and included race and ethnicity [6], educational attainment (highest degrees obtained), [6] yearly family income (greater or less than $40,000) [51], and smoking status (current or not current smoker) [6, 52]. Depression, which is related to cognition [6] and the gut microbiome [53], was measured with the Center for Epidemiological Studies–Depression (CESD), a 20-item questionnaire that measures depressive symptoms and can identify those at high-risk for clinical depression [54]. The CESD was not used to determine eligibility.

### Medication use

Participants brought their medications, supplements, vitamins, and minerals to the baseline visit. Interviewers documented medications taken during the previous 30 days, including dosage and frequency of use. Medications and supplements examined were those shown to be robustly associated with the microbiome (*i*.*e*., estrogens, progesterone, anti-histamines, osmotic laxatives, prebiotics, and probiotics) [52].

### Sensitivity analysis

A sensitivity analysis was performed to determine if the four men in the study sample appreciably altered the gut microbial structure analysis and the differential abundance analysis. This was undertaken as sex is strongly associated with the gut microbiome [52]. To accomplish this, results were compared between the full data set and the full data set without the four men.

### Sample size determination

The sample size was estimated using information from a study [55] in which the authors examined the differences in gut microbial diversity between 25 individuals with AD and 25 without AD.

### Statistical analysis

All variables were inspected for normality. The non-normally distributed variables were hs-CRP, serum LBP, and triglycerides and were log-transformed to achieve normality. For continuous socio-demographic, anthropometric, metabolic, and immunological variables, two-sample t-tests or Wilcoxon rank sum test were used for those normally or non-normally distributed, respectively, and for discrete variables, a chi-square test was used to determine group differences. All the tests were two-sided and *p*<0.05 was considered as statistical significance.

Lastly, to investigate the potential mediating role that the markers of systemic inflammation, oxidative stress, endotoxin translocation and butyrate production might play in connecting cognition to gut microbial taxa, full models were further adjusted, one by one, by these four potential mediators. If, after said adjustment, the model coefficient for MCI status or MoCA (treated as a continuous variable) went from significance to non-significance, the potential mediator was considered a possible mechanistic link connecting the gut microbiome to cognition. The fully adjusted model with MCI as the main predictor included sedentary time, college graduate (yes/no), usage of microbiome-altering and anti-inflammatory medication and DBP. The fully adjusted model with MoCA as the main predictor included college graduate (Y/N), usage of microbiome-altering and anti-inflammatory medication. The statistical analyses were conducted using SAS v9.4 (SAS Institute, Cary, NC).

## Results

### Participant characteristics

Among cases and controls, the mean age was 64.8 years (SD = 5.7 years) and the mean BMI was 36.0 kg/m^2^ (SD = 3.9). Participants were predominately female (93%) and African American (93%, Table 1). Twenty-seven percent were using gut microbiome-associated medications/supplements such as antihistamines or prebiotics. We did not include metformin in the category of gut microbiome-associated medications/supplements because it was not a medication significantly associated with the gut microbiome in the cohort in Falony, et al. [52], which utilized a large, well characterized cohort and considered medication’s effect on the gut microbiome after removing colinear variables from a list of 503 other metadata variables. However, in some cohorts, it has been shown to be associated with the gut microbiome [56, 57]. Nevertheless, metformin usage was similar between cases (n = 2) and controls (n = 4) (Fisher’s exact test: Table Probability = 0.24, p-value = 0.7).

There were a few significant differences between groups. Compared with controls, there were less college graduates among cases (12 vs. 19, p = 0.035). Cases were also less sedentary (14.8 vs. 15.5 hr/day, p = 0.0497) and had higher levels of DBP (83 vs. 79 mmHg, p = 0.054).

Additionally, cases and controls had similarly high levels of hs-CRP, indicative of systemic inflammation [58] and similarly high levels of HbA1c, indicative of pre-diabetes (defined by a HbA1c between 5.7 and 6.4%) [59]. Serum LBP was higher among cases compared to controls (32.7 vs. 26.0 ng/mL, p = 0.08, Table 2), as was *BcoA* abundance (24.1 vs. 24.8 cycles, p = 0.27) (lower cycle number indicates higher abundance). The *BcoA* gene encodes for an enzyme which catalyzes the last step of microbial butyrate synthesis [60] and represents the functional potential of the gut microbiota to produce butyrate [61], an anti-inflammatory [62] short chain fatty acid that can also reduce gut permeability [63].

**Table 2:** Biomarkers stratified by case control status.

Variable	Cases: Probable MCI (n = 30)	Controls (n = 30)	*p*-value
hs-CRP, mg/L (median (IQR))	4.3 (9.3)	5.0 (5.5)	0.28
PGF2α, ng/mL (mean (SD))	0.048 (0.039)	0.054 (0.049)	0.57
8-iso-PGF2α, ng/mL (median (IQR))	0.047 (0.026)	0.048 (0.017)	0.44
Glucose, mg/dL (median (IQR))	93 (10)	93 (16)	0.43
HbA1c, % (mean (SD))	5.8 (0.6)	5.8 (0.7)	0.74
Total cholesterol, mg/dL (mean (SD))	190 (31)	199 (37)	0.33
HDL cholesterol, mg/dL (mean (SD))	59 (17)	66 (17)	0.16
LDL cholesterol, mg/dL (mean (SD))	110 (28)	114 (33)	0.62
Triglycerides, mg/dL (median (IQR))	97 (64)	89 (52)	0.51
Serum LBP, ng/mL (median (IQR))	32.7 (16.3)	26.0 (8.6)	0.08
*BcoA* abundance, cycles until threshold* (mean, SD)	24.1 (1.7)	24.8 (2.9)	0.27

*Lower values represent higher abundance. N = 30 for controls. N = 29 for cases as one case had missing data. *p*-values were determined for all continuous variables by a two-sample t-test, except for 8-iso-PGF2α and glucose, for which the Wilcoxon Rank Sum test was used. *p*-values were determined for all categorical variables by a chi-squared test. Abbreviations: 8-iso-PGF2 α—8-iso-prostaglandin-F2α; BCoA—Butyryl-CoA; hb—hemoglobin; HDL—high density lipoprotein; hs-CRP—high sensitivity C-reactive protein; IQR—Interquartile Range; LBP—lipopolysaccharide binding protein; LDL—low density lipoprotein; MCI—mild cognitive impairment; MoCA—Montreal Cognitive Assessment; PGF2α—prostaglandin-F2α.; SD—Standard Deviation

### Stool microbiome

**Fig 1:**
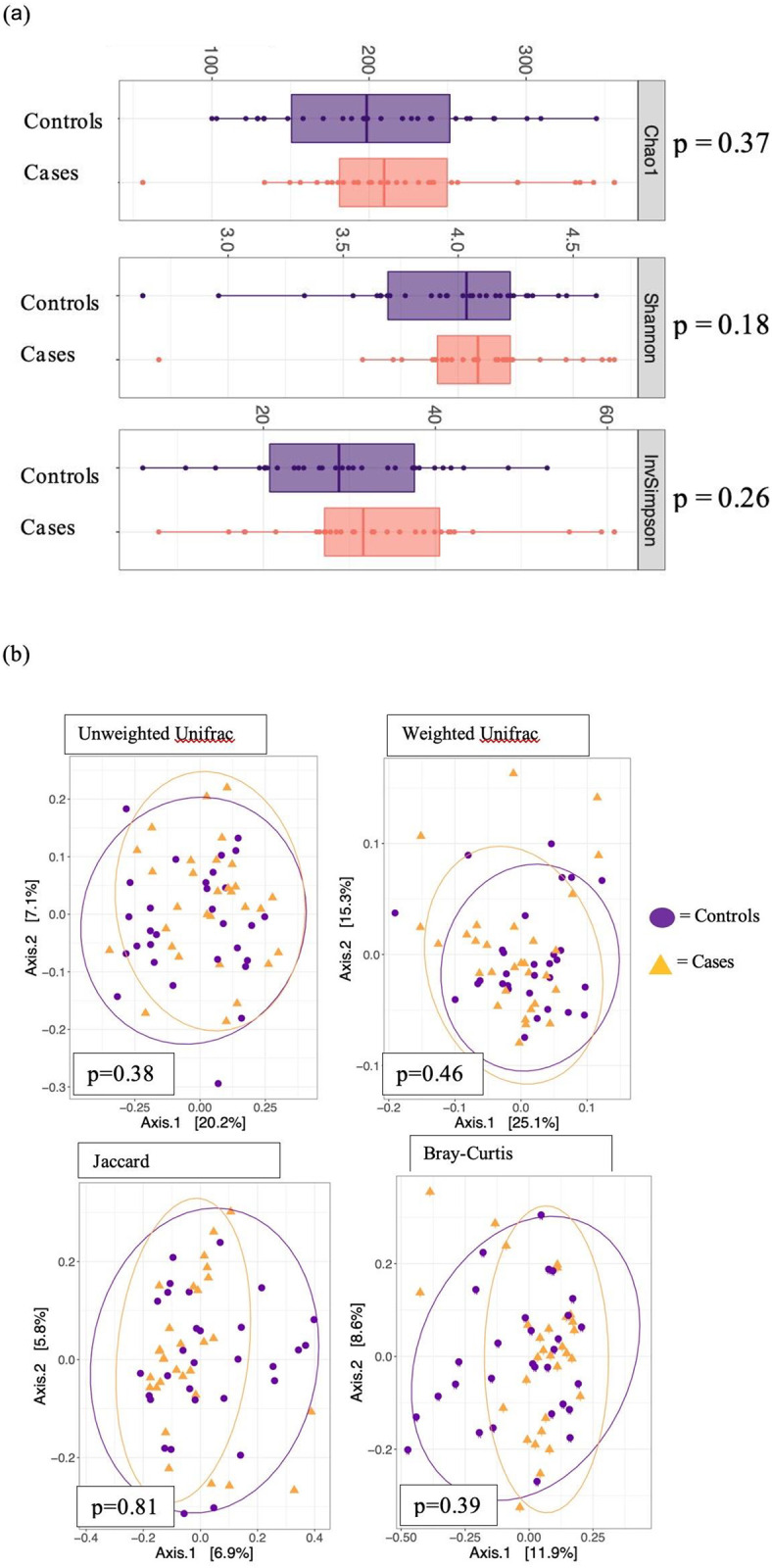
Alpha and beta diversity by case-control status. **(a)** Alpha diversity metrics by case control status—Chao1, Shannon, and Inverse Simpson **(b)** Beta diversity metrics by case control status–PCoA plots of normalized Unifrac distances, unweighted (p = 0.38) and weighted (p = 0.46), Jaccard index (p = 0.81), and Bray-Curtis index (p = 0.39). *p* values correspond to between-group comparisons using PERMANOVA. *p*<0.05 is statistically significant. Cases = yellow triangles and controls = purple circles. Abbreviations: MCI—Mild Cognitive Impairment.

Next, we examined the relationship between cognitive impairment and the gut microbiome. While there was no difference in alpha- and beta-diversity between the cases and controls (p = 0.18–0.81) (see Fig 1), we identified several taxa whose normalized abundance was statistically significant at the amplicon sequence variant level (ASV).

**Fig 2:**
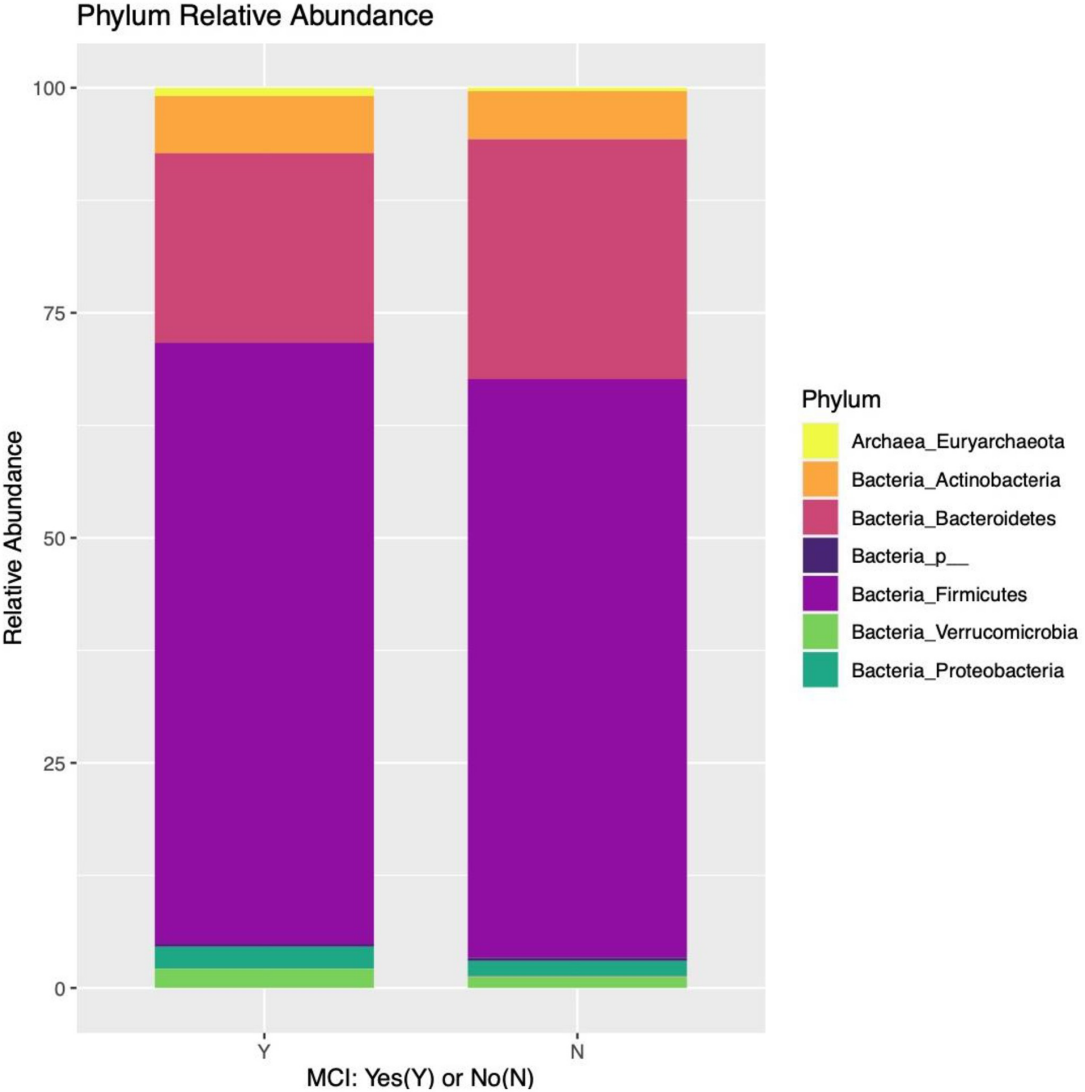
Phyla relative abundance by case-control status.

Fig 2 illustrates the relative abundance of phyla for cases and controls.

**Fig 3:**
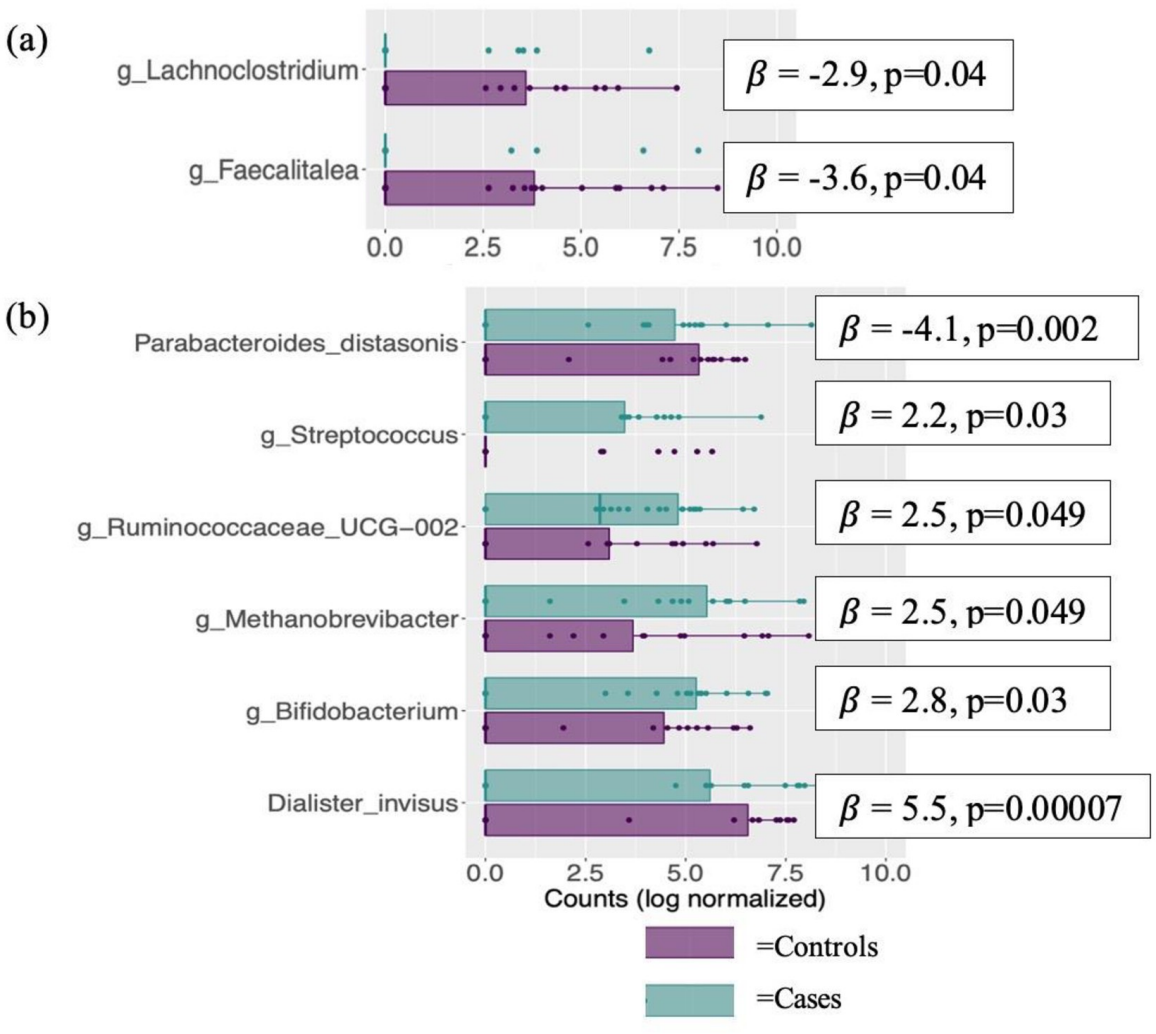
ASV’s differentially abundant between cases and controls. ASV’s differentially abundant between cases = blue squares and controls = purple squares in (a) unadjusted model and a (b) model adjusted by sedentary time, College Graduate (Y/N), usage of microbiome-altering and anti-inflammatory medication and DBP. Abbreviations: ASV—Amplicon Sequence Variant; DBP—Diastolic Blood Pressure; MCI—Mild Cognitive Impairment.

There were no genera significantly associated with MCI status, but we did observe lower levels of ASV associated with the genera *Lachnoclostridium* (*β* = -2.9, p = 0.04) and *Faecalitalea* (*β* = -3.6, p = 0.04) in cases (see Fig 3(a)), yet when we adjusted the model for sedentary time, college graduate (yes/no), usage of microbiome-altering (e.g., antibiotics) and anti-inflammatory medication (yes/no) and DBP, the ASVs associated with genera *Lachnoclostridium* and *Faecalitalea* were no longer significant (p = 0.07 each), but six other ASV’s emerged as statistically significant (higher in cases: *Streptococcus*, *Ruminococcaceae UCG-002*, *Methanobrevibacter*, *Bifidobacterium*, *Dialister invisus*; lower in cases: *Parabacteroides distasonis*) (see Fig 3(b)).

**Fig 4:**
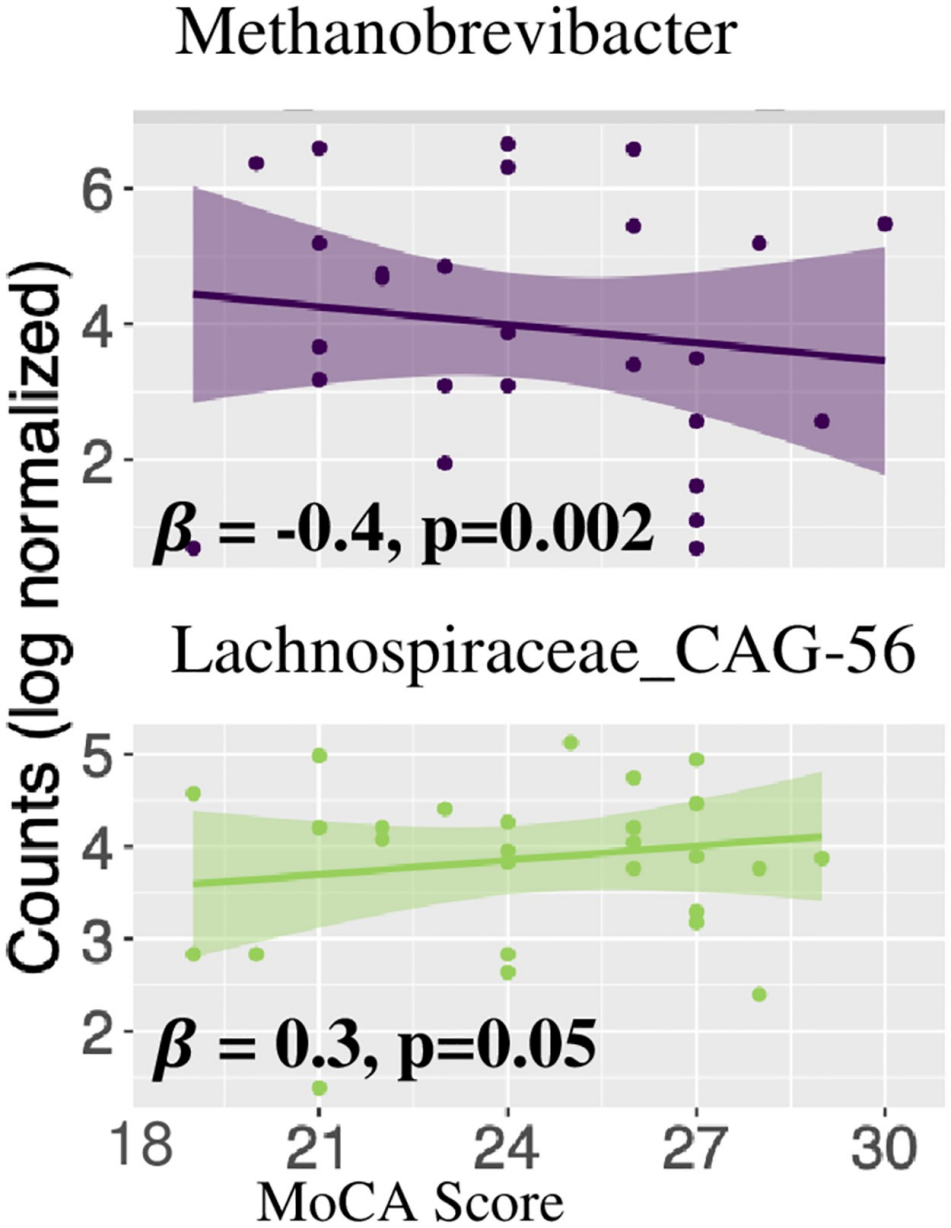
Log normalized counts of genera significantly associated with MoCA score. Scatter plots of log normalized counts of two genera significantly associated with MoCA score in adjusted models. *β*s correspond to beta coefficients from the zero-inflated adjusted linear model. Scatter plots are overlayed with a linear regression line to assist in pattern visualization. Abbreviations: MoCA—Montreal Cognitive Assessment.

We also examined the association between MoCA score and gut microbial composition. Using a zero-inflated general linear model, adjusted by college graduate (yes/no), and usage of microbiome-altering and anti-inflammatory medication, we identified two genera, *Methanobrevibacter* (negatively associated) and *Lachnospiraceae_CAG-56* (positively associated) (see Fig 4).

**Fig 5:**
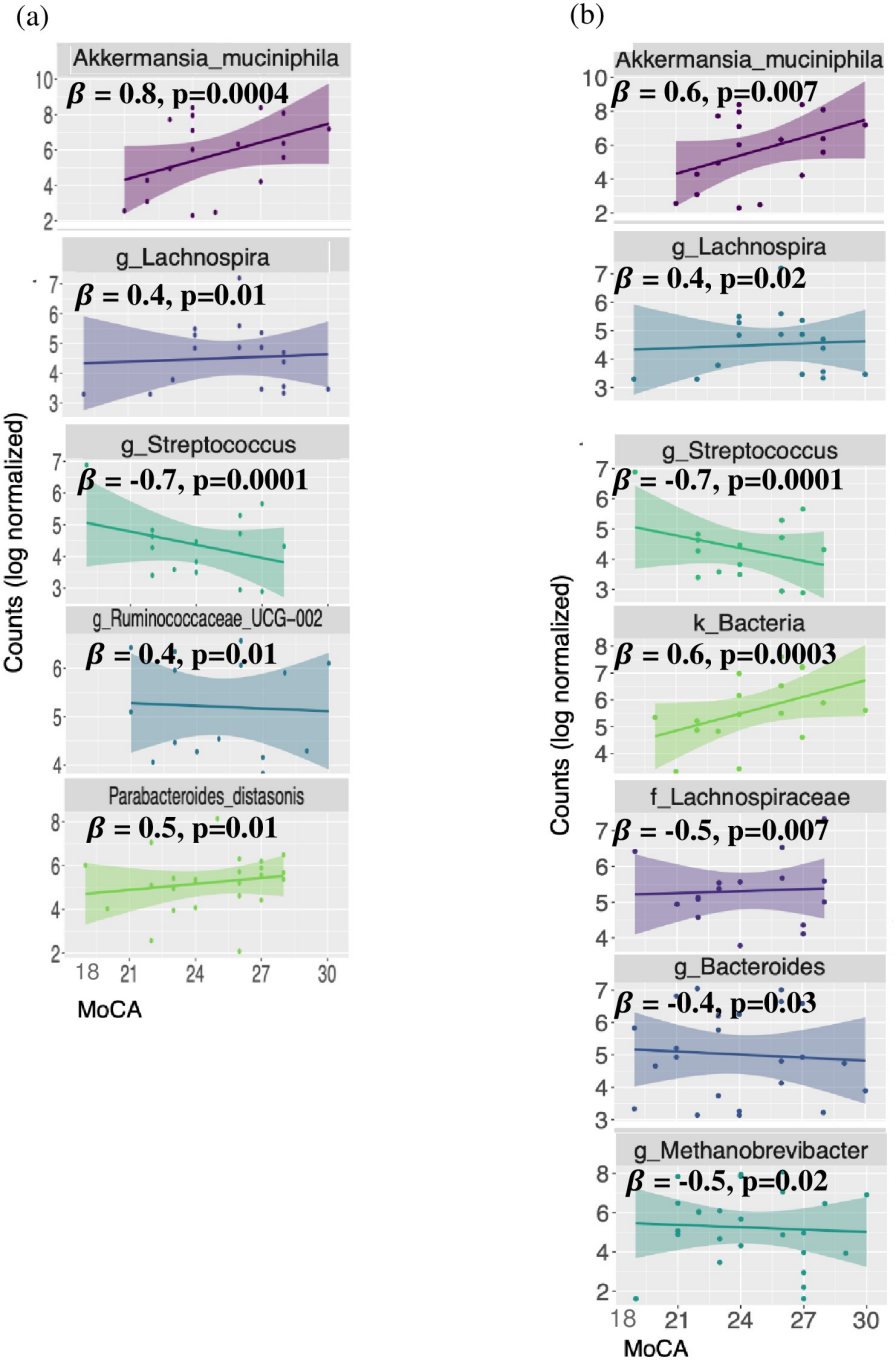
Log normalized counts of ASVs significantly associated with MoCA score. **(a)** Scatter plots of log normalized counts versus MoCA score for the 5 differentially abundant ASVs. The taxonomies shown provide the lowest known taxonomic level. For example, the ASV identified as k_Bacteria was only identifiable down to the kingdom level of Bacteria. *β*’s correspond to beta coefficients from the zero-inflated unadjusted linear model. Scatter plots are overlayed with a linear regression line to assist in pattern visualization. **(b)** Same as part (a) except these ASVs are the differentially abundant ASVs from the adjusted model, which was adjusted by College Graduate (Y/N), usage of microbiome-altering and anti-inflammatory medication. Abbreviations: ASV—Amplicon Sequence Variant; MoCA—Montreal Cognitive Assessment.

In addition, when ASVs were modeled as a function of MoCA score, there were five ASVs (*Akkermansia muciniphila*, *Lachnospira*, *Streptococcus*, *Ruminococcaceae_UCG-002*, *P*. *distasonis*) that were found to be significantly associated with MoCA score in unadjusted models (see Fig 5(a)). In the adjusted model, ASVs associated with genera *Ruminococcaceae_UCG-002* and *P*. *distasonis* were no longer significant, but four additional ASVs emerged as significant, one from unclassified Bacteria, one from unclassified Lachnospiraceae (family), and the species of the genera *Bacteroides* and of the hydrogen-consuming methanogenic archaeon *Methanobrevibacter* (see Fig 5(b)).

*Examining Markers of Systemic Inflammation*, *Oxidative Stress*, *Bacterial Endotoxin Translocation*, *and Butyrate Production Potential as Mediators of the Cognition-Microbiome Relationship*

We next determined if markers of systemic inflammation (i.e., hsCRP), oxidative stress (i.e., PGF2α and 8-iso-PGF2α), bacterial endotoxin translocation (i.e., LBP), and butyrate production potential (i.e., *BcoA*) were possible mediators of the cognition-microbiome relationship by including the above variables in the zero-inflated generalized linear models. Table 3 presents the results of this analysis for ASVs from Figs 3 and 5 whose relationships with case-control status/MoCA score were attenuated by the addition of the mediator. For example, the relationship between *D*. *invisus* and case-control status was attenuated after adjusting for hs-CRP, suggestive of its mediating role. We define attenuation as a change from significance to non-significance for the case-control/MoCA score beta-coefficient. Other relationships were attenuated after adjusting for 8-iso-PGF2α, LBP, and *BcoA* suggestive of their potential mediating role as well.

**Table 3:** Fully adjusted models with MCI* or MoCA** as main predictors.

**Model with MCI as Main Predictor**			
**ASV**	**MCI Coefficient Before Adjustment**	**Hypothesized Mediator**	**MCI Coefficient After Adjustment**
*Bifidobacterium*	*β*_Before_ = 2.8 ± 0.53 (p = 0.03)	LBP	*β*_After_ = 2.48 ±0.6, (p = 0.21)
*Methanobrevibacter*	*β*_Before_ = 2.50 ±0.40 (p = 0.049)	LBP	*β*_After_ = 1.79 ± 0.41 (p = 0.22)
*Streptococcus*	*β*_Before_ = 2.17± 0.53, (p = 0.034)	BcoA	*β*_After_ = 1.66 ± 0.54, (p = 0.16)
*Parabacteroides distasonis*	*β*_Before_ = -4.13± 0.68, (p = 0.002)	8-iso-PGF2α	*β*_After_ = -1.3± 0.71 (p = 0.72)
*Methanobrevibacter*	*β*_Before_ = 2.50 ±0.40 (p = 0.049)	8-iso-PGF2α	*β*_After_ = 2.22 ± 0.42), (p = 0.14)
*Dialister invisus*	*β*_Before_ = 5.5 ±0.65, (p<0.0001)	hs-CRP	*β*_After_ = 0.7 ± 0.5, (p = 0.9)
**Model with MoCA as Main Predictor**			
**ASV**	**MoCA Coefficient Before Adjustment**	**Hypothesized Mediator**	**MoCA Coefficient After Adjustment**
*Methanobrevibacter*	*β*_Before_ = -0.47± 0.07 (p = 0.02)	LBP	*β*_After_ = -0.344 ± 0.08 (p = 0.19)
*Methanobrevibacter*	*β*_Before_ = -0.47± 0.07 (p = 0.02)	BcoA	*β*_After_ = -0.6 ± 0.08 (p = 0.0013)
*Methanobrevibacter*	*β*_Before_ = -0.47± 0.07 (p = 0.02)	8-iso-PGF2α	*β*_After_ = -0.41 ± 0.08 (p = 0.09)

Effect on Fully Adjusted Model Coefficients After Further Adjustment For hs-CRP, Oxidative Stress, LBP, or BcoA.

*Fully adjusted model with MCI as main predictor was adjusted for sedentary time, college graduate (yes/no), usage of microbiome-altering (e.g., antibiotics) and anti-inflammatory medication (yes/no) and DBP.

**Fully adjusted model with MoCA as main predictor was adjusted for college graduate (yes/no), usage of microbiome-altering (e.g., antibiotics) (yes/no) and anti-inflammatory medication (yes/no).

Shown are coefficients for MCI status and MoCA score before adjustment and after adjustment for potential mediator variables representing biological mechanisms connecting the gut microbiome with cognition. If a coefficient was *not* attenuated as evidenced by a shift from significance to non-significance, it is not shown. Abbreviations: 8-iso-PGF2 α—8-iso-prostaglandin-F2α; BCoA—Butyryl-CoA; hs-CRP—high sensitivity C-reactive protein; LBP—lipopolysaccharide binding protein; MCI—mild cognitive impairment; MoCA—Montreal Cognitive Assessment.

Lastly, a sensitivity analysis was also performed excluding the four males, and the results remained unchanged (results not shown).

## Discussion

This case-control study compared the gut microbiome between those with and without probable MCI in at risk population, African American women. We determined that while the overall community structures did not differ between cases and controls, several genera and species did. Other studies have been performed in predominantly European ancestry population [21] and Chinese cohorts [64–67]. Of note, this is the first study, to our knowledge, to include predominantly female African American participants, a population that has a higher incidence of dementia than most other racial and ethnic groups in the U.S. [68]. The differences in the gut microbiome found in our study between those with and without probable MCI were independent of several possible confounders including education, use of microbiome-altering medications, antibiotics and physical inactivity. Additionally, the relationship between microbial taxa and probable MCI was shown to be potentially mediated through several biological mechanisms, including systemic inflammation, oxidative stress, microbial butyrate production potential, and systemic bacterial endotoxin translocation, adding plausibility to a gut microbiome-MCI relationship.

Similar to the prior studies, we found no significant differences in the gut microbial diversity between participants with and without probable MCI [21, 64–67]. We also did not detect differences in community structure, as measured by beta-diversity. However, several researchers found that healthy controls presented a distinct community structure than cases with MCI [64, 67], indicating a microbial signature of MCI [65, 66], Just one small sample size study (n = 17) did not detect a significant difference in beta-diversity [21]. Different from prior studies, we matched on co-variates that strongly regulate the gut microbiota including age, BMI and sex, which could have led to no distinction in the microbial community structures between cases and controls.

We identified several ASV that were associated with MCI. For instance, *P*. *distasonis* was depleted in cases compared with controls and is reduced in other neurological disorders that negatively impact cognition, *i*.*e*., autism spectrum disorder [69]. When we further adjusted the model for oxidative stress, *P*. *distasonis* was no longer significantly different between cases and controls suggesting *P*. *distasonis* may partially relate to cognition through oxidative pathways, although this would need to be confirmed in a laboratory setting. Only one prior study investigated species-level differences between those with and without MCI, and the authors of that study did not identify *P*. *distasonis* as differentially abundant [67]. Nevertheless, given this bacterium’s association with cognitive disorders and a potential mechanism for them, it should be a focus of future studies, perhaps using more accurate and quantitative detection methods such as qPCR of a gene specific to *P*. *distasonis*.

*D*. *invisus* is another ASV whose abundance was significantly higher in cases compared to controls, and it may relate to cognition via systemic inflammatory pathways. *D*. *invisus* has been studied less in the context of the intestinal microbiome than in the context of the oral microbiome and periodontal disease [70]. One study supports the proposition that *D*. *invisus* in the gut is dead, or at least not proliferating, given it exhibited no metatranscriptomic activity there [71]. This suggests that *D*. *invisus* abundance in the gut could be a proxy for abundance in the oral cavity, where it is known to be related to periodontal disease [70], which is itself associated with CRP [72]. Moreover, periodontal disease is emerging as a potential risk factor for cognitive decline [73]. Thus, the relationships seen in our study between *D*. *invisus* and cognition may actually be reflective of the oral microbiome, not the gut microbiome. Oral health history was not part of our health questionnaires but should be considered in future studies.

In our study, the ASV showing the most consistent relationship with cognition was *Methanobrevibacter* a hydrogen-consuming methanogenic archaeon. *Methanobrevibacter* was higher in relative abundance in cases compared with controls and in those with lower MoCA score when it was examined as a continuous variable and remained differentially abundant in fully adjusted models. Furthermore, its relationship with cognition may be partially explained by oxidative stress, bacterial endotoxin translocation, and butyrate production potential. *Methanobrevibacter* consumes hydrogen which has been shown to quell brain oxidative stress in a rat model, after either oral administration of hydrogen-rich water or inhalation of hydrogen gas [74, 75]. Thus, an increasing abundance of *Methanobrevibacter* may reduce concentrations of hydrogen, lowering the capacity of the brain to handle oxidative stress, and ultimately leading to impaired cognition [76].

Though *Methanobrevibacter* is a genus of the *Archaea* kingdom and therefore does not contain lipopolysaccharide [77] (LPS) (a component of which is endotoxin [78]), this genus has been shown to co-occur with bacterial genera that positively correlate with liposaccharide binding protein (LBP) [79]. Alternatively, LBP is a positive acute phase protein and may be increased due to stimuli other than LPS [80]. Measuring LPS in future studies may help confirm or debunk the relationship between *Methanobrevibacter* and LBP.

Interestingly, when controlling for *BcoA* abundance, the inverse relationship between *Methanobrevibacter* and MoCA modeled as a continuous variable was further strengthened. This suggests that butyrate may mitigate the detrimental relationship that *Methanobrevibacter* has with cognition. This is biologically plausible given that this archaeon improves the fermentative capacity of butyrate producers by consuming hydrogen that would otherwise impair butyrate production [81].

Another interesting finding is the positive association between *Akkermansia muciniphila* abundance and MoCA when modeled as a continuous variable. In a double-blinded, placebo-controlled, randomized trial *A*. *muciniphila* supplementation reduced levels of inflammation, insulin resistance, and plasma levels of LPS compared to placebo [82]. Inflammation and insulin resistance are associated with cognitive decline [19, 83] and LPS can drive systemic inflammation [15]. Thus, the lower abundance of *A*. *muciniphila* in the guts of those with MCI may partially explain their likely impaired cognition.

There were only two differentially abundant ASVs from prior studies that were also differentially abundant between cases and controls in our study mapped to the genera *Bacteroides* and *Ruminococcaceae_ucg_002*. In Guo et al., the genus *Bacteroides* was higher in controls and positively correlated with MoCA whereas in our study an ASV identified as the same genus was lower in controls and negatively correlated with MoCA. In Li, et al., in contrast to our study, *Bacteroides* was higher in controls. In agreement with our study, *Bacteroides* was lower in controls in Liu, et al. In only one other study was *Ruminococcaceae_ucg_002* found to be differentially abundant, and there was agreement with our study—*Ruminococcaceae_ucg_002* was increased in cases. Lastly, in a study by Meyer, et al. that associated the gut microbiome with cognitive scores in 270 middle-aged, African Americans, the genera *Sutterella* and *Parasutterella* were negatively and positively associated, respectively, with MoCA in adjusted models. These two genera were not associated with MoCA in our adjusted models. This discrepancy may have been due to the larger sample size in Meyer, et al., or the different covariates used in modeling [84]. Given this apparent lack of consensus across the few studies examining the gut microbiome in those with and without probable MCI, it is premature to generalize as to what taxa are uniquely related to MCI. More studies are needed using more specific analytical methods, such as shotgun sequencing (metagenomics) and RNA-seq (meta-transcriptomics) as closely related microbial organisms even within the same species, can have very distinct metabolisms and functions [85].

Our study is a strong step forward, though its findings should be interpreted in light of its strengths and limitations. A major strength of our study is the matching that we performed between cases and controls. It gave us a more homogenous group of participants that allowed for us to exclude possible confounders from our regression models, making those models more robust. Another strength was our comprehensive assessment of possible covariates, such as medication usage. This allowed us to more fully elucidate the unique contribution of the gut microbiome to probable MCI status. A third strength is that our subjects were primarily African American, a population that is disproportionately impacted by dementia but less studied [68] Lastly, we measured potential mediating biomarkers including CRP, oxidative stress markers, an indirect measure of bacterial endotoxin translocation, and a gene associated with microbial capacity to produce butyrate to allow for a deeper understanding of how the gut microbiome may be related to cognition.

Our study is not without limitations. Because of the case-control design, the possibility of residual confounding and reverse causation cannot be excluded. Though we measured several potential confounders and adjusted for the ones that were different between cases and controls, we may have failed to account for others including stool consistency (e.g., firm or loose) [86]. Hard stool has been shown to be correlated with higher levels of *Methanobrevibacter* [86]. Because dietary fiber increases *Bifidobacterium* concentrations in the gut [87], and cases had greater relative abundance of an ASV assigned to the *Bifidobacterium* genus we examined if dietary fiber consumption post-hoc was different between cases and controls. We found that the log of dietary fiber per 1,000 kcal was not significantly different between cases and controls (2.5 g/1000kcal/day v. 2.4 g/1000kcal/day, respectively, p = 0.7). Regarding reverse causation, some of the gut microbiome-MCI associations seen in this study may reflect a process whereby decreased cognition leads to changes in health behaviors that in turn impact the microbiome.

Another limitation is that we did not clinically diagnose MCI in our participants, but instead used a screener, the MoCA, with the more common MoCA cut point of <26 to classify probable MCI. While highly accurate in initial studies (sensitivity and specificity of 90% and 87%, respectively [27]), a recent study showed that the <26 cut point had a higher false negative rate compared to a <24 cut point in a predominately female African American cohort [88]. Thus, our use of a <26 cut point vs. <24 cut point may have led to even more misclassification of cases and controls, although the cut-point we used is similarly accurate at 76% [88]. Furthermore, though MCI is prodromal to dementia, some individuals do not transition to dementia and some even revert to normal cognition [5]. Hence, the findings from the current study may not fully apply to those with MCI who do eventually convert to dementia.

Lastly, the gut microbiome can change significantly as a result of what one consumes the day prior to sampling [89]. We only measured one year of habitual diet through a FFQ thus potentially missing a significant recent source of variability in the gut microbiome. A 24-hour dietary recall may have been more appropriate and may have shown that recent diet was different between cases and controls. However, we did use a Med Diet screener to assess eligibility on the same day as we administered the FFQ, and this screener includes questions regarding food intake from the prior week. The scores were very similar between cases (4.7 (±1.4)) and controls (4.4 (±1.3)), p = 0.38), suggesting that recent dietary intake was similar between groups.

To conclude, our study investigated the differences in the gut microbiome among obese older predominately female African American participants with and without probable MCI. We have shown that there are microbial distinctions between those with and without probable MCI and that the relationship between these differentially abundant taxa and MCI may be partially explained by oxidative stress, systemic inflammation, and microbial metabolites. Although our findings should be interpreted with caution, taken together with the existing U.S. and Asian based studies, characterizing the gut microbiome may have important clinical implications by serving as a tool to identify those with MCI who will transition to dementia. Moreover, the gut microbiome may be used as a therapeutic target to arrest or slow the progression of MCI, and ultimately the development of AD. Future studies should measure additional host-microbial interactions, such as microbial and microbial-regulated metabolites (e.g., other SCFAs, gamma-butyric acid) and microbial gene transcripts to more fully understand how the gut microbiome is different vis a vis disease status. Lastly, fecal transplant studies from human cases and controls using germ-free animal models would further elucidate mechanisms and provide causal evidence that the gut microbiome plays an integral role in cognitive health.

## Supporting information

S1 Table Deidentified participant demographic, socioeconomic, and biochemical data. (CSV)

S1 Appendix R Code for gut microbiome analysis. (R)

S2 Appendix SAS code for all other analysis. (SAS)
